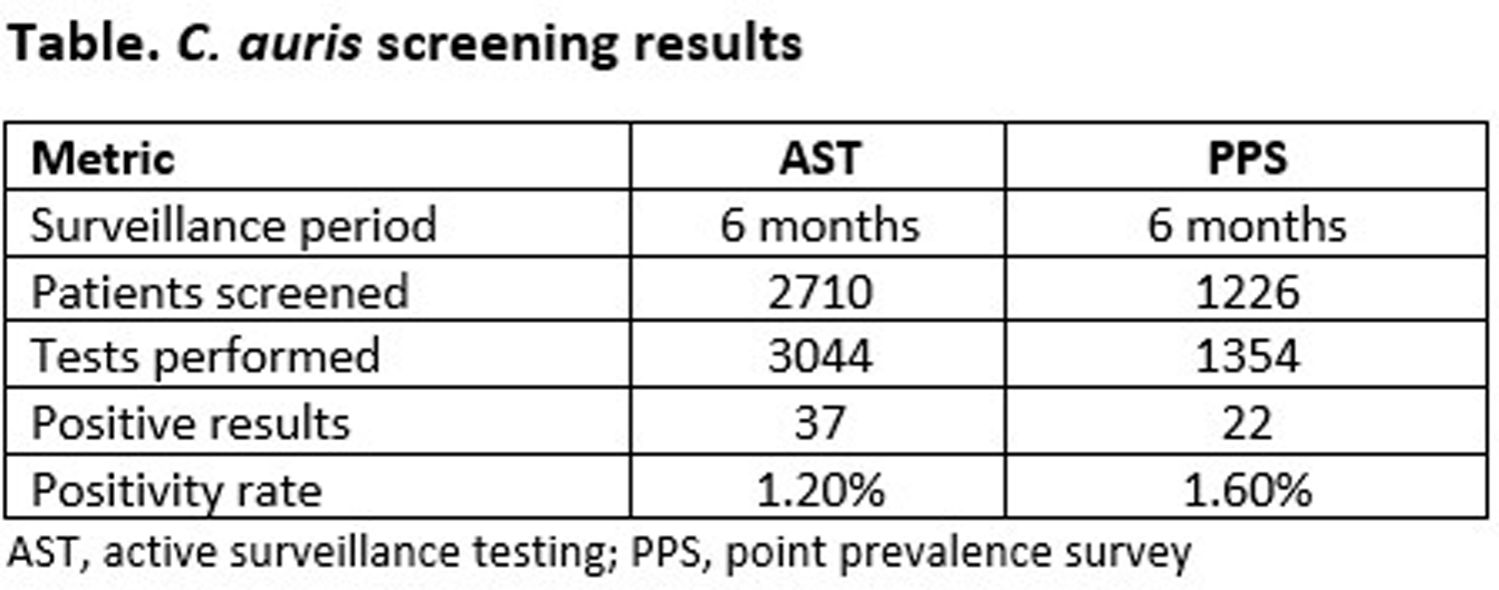# 259 Clostridioides difficile Whole Genome Sequencing Does Not Support Transmission from Environmental Contamination by Prior Room Occupants

**DOI:** 10.1017/ash.2026.10733

**Published:** 2026-06-23

**Authors:** Geehan Suleyman, Anita Shallal, Kathy Callahan, David Schwartz, Linda Vergilio-Panek, Robert Tibbetts, Linoj Samuel

**Affiliations:** 1 Henry Ford Health; 2 Henry Ford Hospital

## Abstract

**Background:** Candida auris, an emerging multidrug-resistant yeast, spreads rapidly in healthcare settings and is associated with difficult-to-control outbreaks. Prevalence has risen significantly in the Metro Detroit area, driven by interfacility transfers and inconsistent communication of colonization status. Patients can remain colonized for prolonged periods and shed the organism into the environment, where it persists for months. Active surveillance testing (AST) can support earlier detection and containment. We describe the implementation of a C. auris AST program across a multi-hospital health system. **Methods:** Screening for C. auris colonization can be either prevention?based or response?based. Response?based strategies, such as point prevalence surveys (PPS), are recommended by the Centers for Disease Control and Prevention when a new case is identified. Prevention?based AST is also recommended to detect cases early among high?risk groups. In April 2025, we launched a C. auris AST program across five hospitals in Southeast Michigan. Composite axilla–groin swabs were tested by real?time polymerase-chain reaction (PCR). High?risk patients were defined as those presenting from long?term acute care hospitals, skilled nursing facilities, subacute rehabilitation facilities, or inpatient rehabilitation centers who required ED admission or transfer from another healthcare facility (including internal transfers) and had one or more of the following: indwelling devices (e.g., endotracheal or tracheostomy tubes), chronic or non?healing wounds, or colonization/infection with multidrug?resistant or carbapenem?resistant organisms. Upon order signing in the electronic health record, an alert prompted providers to order C. auris PCR and initiate contact precautions. Patients were retested upon readmission. PPS were also performed per state health department recommendations following identification of new cases. **Results:** During the 6?month surveillance period, 2,710 unique patients underwent AST, with 3,044 total tests completed. Of these tests, 969 (31%) were ordered in the emergency department at the time of admission. Overall, 37 patients (1.2%) screened positive for C. auris colonization (Table). During the same period, 1,354 tests were performed through 77 unit?based PPS, yielding 22 positive results (1.6%). **Conclusion:** Although AST was implemented as a prevention?based strategy, PPS remained necessary for identifying additional cases. Positivity rates were low across both approaches, yet each required substantial coordination, staff time, and laboratory resources. These findings highlight the need to balance the benefits of early detection with operational demands. Alternative approaches beyond widespread screening should be explored to optimize resource utilization while maintaining effective C. auris prevention and control.

**Figure:**